# Cavity-enhanced metallic metalens with improved Efficiency

**DOI:** 10.1038/s41598-019-57337-3

**Published:** 2020-01-15

**Authors:** Hanmeng Li, Bin Fang, Chen Chen, Shining Zhu, Tao Li

**Affiliations:** National Laboratory of Solid State Microstructures, Key Laboratory of Intelligent Optical Sensing and Integration, Jiangsu Key Laboratory of Artificial Functional Materials, College of Engineering and Applied Sciences, Nanjing University, Nanjing, 210093 China

**Keywords:** Metamaterials, Sub-wavelength optics, Metamaterials, Sub-wavelength optics

## Abstract

Metasurfaces are made of subwavelength nanoantennas with a flat, ultrathin architecture, and strong capability in manipulating the propagation of light by flexible modulations on its phase, amplitude, and polarization. Conventional metallic metalenses always suffer from its low efficiencies due to large intrinsic loss. Here, we demonstrate a cavity enhanced bilayer metalens composed of aluminum nanobars and its complementary structures. The focusing and imaging experiments definitely show an improved efficiency of such kind of bilayer metalens compared with its single layer counterpart. Detailed theoretical analyses based on full-wave simulations are carried out with respect to different cavity lengthes and working wavelengths, which reveals that the improvement rightly attributes to enhanced cavity mode. Our design will not only improve the working efficiency for metalens with simplified manufacturing procedure, but also indicates more possibilities by employing the metal as electrodes.

## Introduction

Metasurface is a kind of two-dimensional (2D) arrayed ultrathin structure with subwavelength unit cells^1^, which is of strong capability to manipulate the phase, amplitude, polarization of light within a ultrathin film and thus arrested remarkable attentions in recent years^2–16^. Plenty of functionalities based on metasurfaces have been demonstrated, such as optical vortex plates^2,7^, holograms^16–22^, color filters^23^, and so on. By utilizing a conical phase profile, metasurface can work as a lens (*i.e*., metalens), which is capable of focusing incident light within a very compact dimension. Metallic metalens has shown its strong capability in beam focusing with a very thin layer^5,13^. However, the high intrinsic losses of metal in optical wavelengths (especially in the visible range) greatly limit the metalens working efficiency and prevented its real applications. Although this challenge can be circumvented by using reflective-type metasurfaces^18^, transmission-type devices have a much wider range of applications. More recently, people have developed all-dielectric metasurfaces to obtain high efficiency in transmission scheme, and important progresses have been achieved in metalens imaging^24^, efficient holography^25^, achromatic design^26–31^, and so on. Nevertheless, there are still challenges in nanofabrication for large scale devices according to requirement of high-aspect-ratio nanostructures, which are essential in dielectric metasurface designs. In addition, all-dielectric design somewhat restricts applying external electric field, which is a common means in tunable devices with electro-optical effect. Therefore, it would be applausive to develop metallic metasurfaces with improved transmission efficiency, where the metallic layer would possibly work as electrodes as well. However, there are only few studies on improving the efficiency of metallic metasurfaces and most of them are limited in reflection-type devices^18,19,32^.

In this letter, we propose a transmission-type metallic metalens with a cavity inside to improve the working efficiency significantly both in simulation and experiments. It is clearly observed in the improvement of diffractive efficiency with strong focusing intensity and enhanced imaging contrast despite of the decrease in total transmission. The cavity plays an important role with local field enhancement that gives rise to the ultimate improved efficiency. Moreover, this bilayer metalens can further reduce the processing in nanofabrications compared with the conventional lift-off approach, and hold the advantage for massive production.

## Results and Discussions

**Figure 1:**
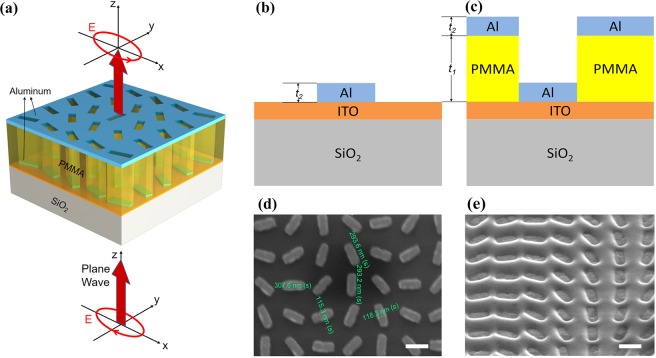
(**a**) Schematics of part of the bilayer metalens (BLM) under circular polarized illumination with cross-polarization analysis after transmission. (**b**) Side section view of the unit cell of the (**b**) BLM and (**c**) conventional metalens only composed of aluminum. (**d**,**e**) SEM micrographs of the two kinds of metalens corresponding to (**b**,**c**), respectively (scale bars = 300 nm).

Figure 1(a) schematically shows the bilayer metalens (BLM) with a circular polarized illumination, where a polymethyl methacrylate (PMMA) spacer is designed with nanohole array on a silica substrate, and aluminum rectangular nanobars is filled inside nanoholes and their complementary structure on the top surface. These Al nanobars are rotationally arranged according to the geometric phase (*i.e*., Pancharatnam–Berry (PB) phase) design^2^. To more clearly show the structural parameters, the side section of the unit cell is shown in Fig. 1(c). Compared with the conventional metallic metalens (Fig. 1(b)) only with metal nanobar/nanodisc on a dielectric substrate, the proposed BML can be regarded as a staggered cavity. The corresponding scanning electron microscope (SEM) images of two samples are shown in the Fig. 1(d,e), which are fabricated by e-beam lithography (ZEISS ULTRA 55). In fact, this bilayer sample (Fig. 1(c,e)) can be regarded as partially prepared product compared with the conventional metasurfaces (Fig. 1(b,d)), since it lacks the final lift-off procedure. To function like a focusing lens, the phase profile *φ* (*x*, *y*) of the metalens is designed as

1 $$\varphi (x,y)=\frac{2\pi }{{\lambda }_{0}}(f-\sqrt{{x}^{2}+{y}^{2}+{f}^{2}}).$$

Here, *λ*_0_ is the design wavelength and in this work we choose the 632.8 nm, *x* and *y* are the coordinates of each nanobar, and *f* is the focal length. This phase profile is imparted via rotation of each nanobar at a given coordinate (*x*, *y*) by an angle *θ* (*x*, *y*). In the case of right-handed circularly polarized incident light, these rotations yield a phase shift as *φ* (*x*, *y*) = 2*θ* (*x*, *y*), accompanied by polarization conversion to left-handed circularly polarized light. Thus, each nanobar at (*x*, *y*) is rotated by an angle

2 $$\theta (x,y)=\frac{\pi }{{\lambda }_{0}}(f-\sqrt{{x}^{2}+{y}^{2}+{f}^{2}}).$$

Both metalenses with the same diameter of 80 μm and focal length of 120 μm (NA = 0.32) were fabricated by electron-beam lithography (EBL), where the PB phase designed metalens patterns of the PMMA were spin-coated on an ITO coated quartz substrate. The thickness of the PMMA layer is 100 nm (as *t*_1_ in Fig. 1(c)), and electron beam evaporation is subsequently used to deposit aluminum onto the developed resist to form the aluminum nanobars. The thickness of aluminum is 30 nm (referred as *t*_2_), and so does the nanobar thickness. The width *W*, and length *L* of the nanobar here we choose are 120 nm and 300 nm respectively with the periodically arranged unit cell dimensions (*S* = 400 nm). Aluminum is chosen for its relatively low loss in the visible region. For the conventional metallic metalens, a lift-off step is needed to strip the PMMA surroundings with the Al nanobars left only. But in the BLM design, we do not need to conduct the lift-off, which means the deposition of metal is the last step of our fabrication to obtain the bilayer structure. By cutting off the lift-off step, we can not only save much time in fabrication but also avoid the structural fluctuations induced in the last process.

**Figure 2:**
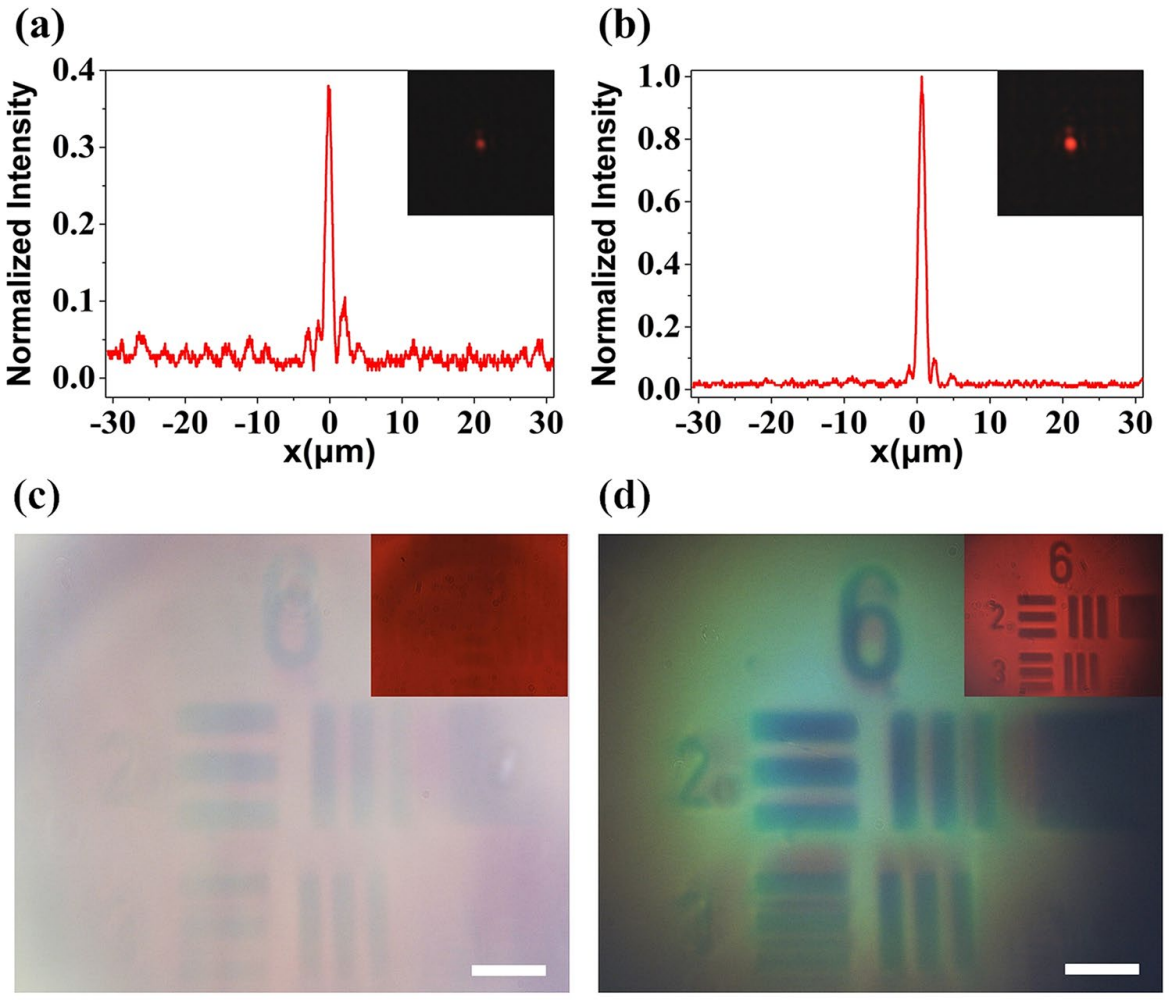
(**a**,**b**) Measured cross-sections of the focal spots of the conventional metalens and the BLM, respectively. The two inserts are the corresponding images of focal spot captured by CCD. (**c**,**d**) Images of 1951 USAF resolution test chart taken from the conventional metalens and the BLM with white light illumination (scale bar = 7 μm). The two inserts are the corresponding results of monochromatic images after 630 nm-wavelength filter, respectively.

Figure 2(a,b) show the cross-sections of the focal spots that are obtained for the conventional metalens and BLM at the design wavelength (*λ*_0_ = 632.8 nm) in our experiments, from which we obtained the full-widths at half-maximum (FWHM) of focal spots are 1.1 μm and 1.05 μm, respectively. The corresponding focal spots images through two metalenses are shown in the insert figures of Fig. 2(a,b). Besides smaller and more symmetric focal spot, the BLM sample gives rise to a much stronger intensity (~2.63 times enhancement) than the conventional one at the same exposure time of the camera with the same illumination. Moreover, the 1951 United State Air Force (USAF) resolution test chart was used as the imaging target, and was illuminated by a halogen white-light source. Figure 2(c,d) show the results from the conventional metalens and BLM. It is clearly shown that image contrast and signal-to-noise ratio (SNR) via BLM is much better than the conventional one, thought it has higher total transmittance. It is further confirmed by the monochromatic image quality as filtered at 630 nm wavelength. The image via conventional metalens even disappear according to its low efficiency, while that of BLM keeps clear observation, as shown in the inset figures in Fig. 2(c,d). The details in optical setups and measurements are provided in Methods.

**Figure 3:**
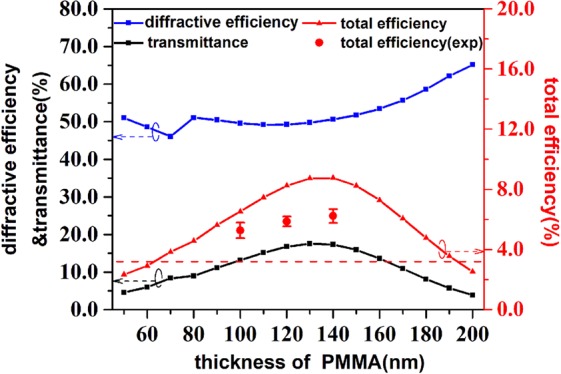
Diffractive efficiency, transmittance (left label), and total efficiency (right label) of the BLM with respect to different thickness of PMMA (related to the cavity length). Here, the small symbols with connection lines are the simulation results, and the larger symbols with error bars are the experimental data. The red dashed horizontal line represents the simulated total efficiency of the conventional single layer metalens.

To quantitively analyze the performance, we defined a diffractive efficiency as

3 $${\eta }_{d}=\frac{{I}_{\sigma -}}{{I}_{\sigma +}+{I}_{\sigma -}},$$

where *I*_*σ* +_ and *I*_*σ* −_ represent the intensity of the detected beam with the same and cross circularly polarization states, respectively. Then, we obtain the total focusing efficiency as

4 $${\eta }_{t}={\eta }_{d}\cdot T,$$

where *T* is the total transmittance of the metalens. Obviously, the total focusing efficiency of our new designed structure is much higher than the conventional one. In the BLM structure, there are several parameters affect the focusing efficiency. Among them, the separation thickness between the two metal layers is considered as a major contribution to the cavity effect that strongly affect the diffractive efficiency and the transmittance. To figure out the cavity effect on the efficiency of the BLM, we employed a commercial software (Lumerical FDTD Solutions) for the full-wave simulations. In simulations, we choose the same parameter as obtained in experiments, say, the thicknesses of the two aluminum layers (*t*_2_) are both 30 nm, the period of the unit cell (*S*) is 400 nm and each nanobar dimension of *W* = 120 nm and *L* = 300 nm. We calculated the diffractive efficiency *η*_*d*_, transmittance *T*, and the total efficiency *η*_*t*_ with respect to different thicknesses of PMMA (*t*_1_), as the results shown in Fig. 3. It is seen that though the transmittance is considerable low (<18%), the diffractive efficiency is relatively high (>45%) in the whole range. As the thickness of PMMA increases from 50 nm to 200 nm in our simulation, both the transmittance *T* and the total efficiency *η*_*t*_ exhibit a maximum when the *t*_1_ ≈ 140 nm. While the *η*_*d*_ undergoes a nearly inverse trend, which changes very little when *t*_1_ < 140 nm.

Compared with the conventional metalens whose diffractive efficiency *η*_*d*_ is only at the level of 5% in our simulation (not shown in Fig. 3), BLM has a much higher diffractive efficiency *η*_*d*_ (the lowest one is 46%). Although the structure of BLM may decrease the transmittance *T*, the improvement of the diffractive efficiency *η*_*d*_ can make up for it and exceed. The red horizontal dotted line represents the value of the total efficiency *η*_*t*_ of the conventional metalens (*η*_*t*_ = 3.2%) while that of BLM is higher when the thickness of PMMA ranges from 70 nm to 190 nm. The maximum efficiency at *t*_1_ ≈ 140 nm (*η*_*t*_ = 8.8%) is about 2.75 times improvement. Our experimental data is also consistent with calculation, as the symbols of red solid circles shown in Fig. 3, where the total efficiency is not as high as the simulation but still reaches an improved level (~6%). According to our experiments with same condition, the conventional single-layer metalens only has 2.31% in total efficiency. Thus, this cavity-enhanced double-layer metalens definitely has a considerable improvement in the focusing efficiency.

**Figure 4:**
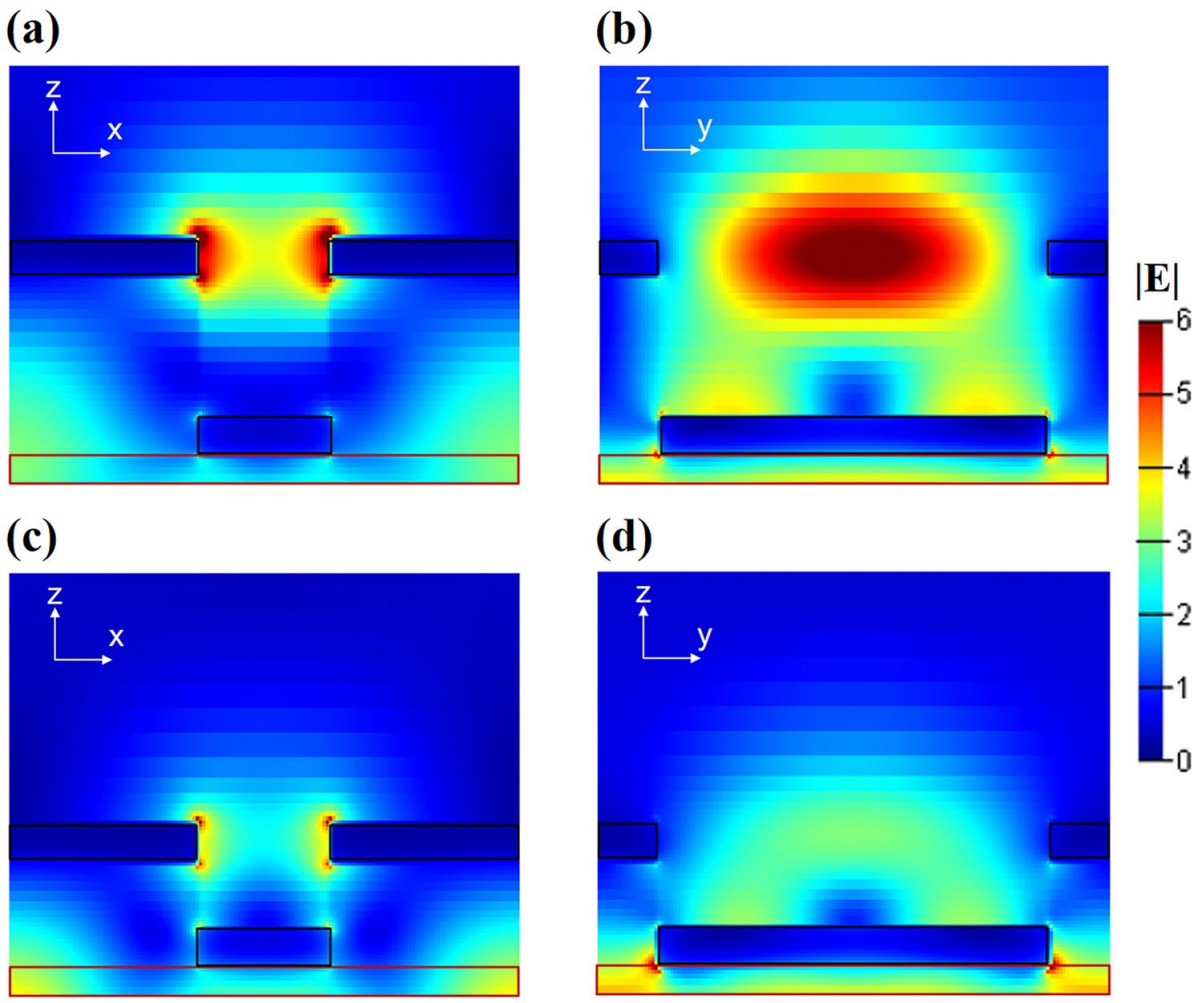
(**a**,**b**) Simulated E-field distribution in the BLM with a period of 400 nm and a separation thickness of 140 nm (optimal) in x-z and y-z planes, respectively, as excited by a circular polarized illumination at *λ*_0_ = 632.8 nm. (**c**,**d**) The corresponding simulated results of the un-optimized cavity (t_1_ = 80 nm), where the E-field is much weaker than that in optimized case. All the electrical field are normalized in same scale.

To understand the cavity enhancement mechanism of the BLM, we choose the optimized parameter (*t*_1_ ≈ 140 nm) case with another un-optimized one (*t*_1_ ≈ 80 nm) for detailed investigations. Figure 4 displays their field distributions in x-z and y-z planes, revealing the characteristics of cavity mode. It is clearly shown that optimized structure exhibits a much stronger field excited at the long-edges of the upper aluminum hole (see Fig. 4(a)) compared to the un-optimized one (see Fig. 4(c)), which gives rise to the improved diffractive efficiency. Indeed, the proper separation thickness (*t*_1_ ≈ 140 nm) should account for a complete cavity mode inside the hole between two metal layers. Figure 4(b) clearly show the cavity mode property in x-z dimension, where we can find a “standing-wave” appearance centered at the upper aluminum layer that should accommodate a resonant mode with wavelength of fourfold the cavity length. In our simulation and experiments, the light wavelength is 632.8 nm, which would correspond to the cavity length of 158.2 nm. Considering our structure, the cavity length of the optimized case is *t*_1_ + *t*_2_/2 = 155 nm, agreeing extremely well with the prediction of cavity model.

**Figure 5:**
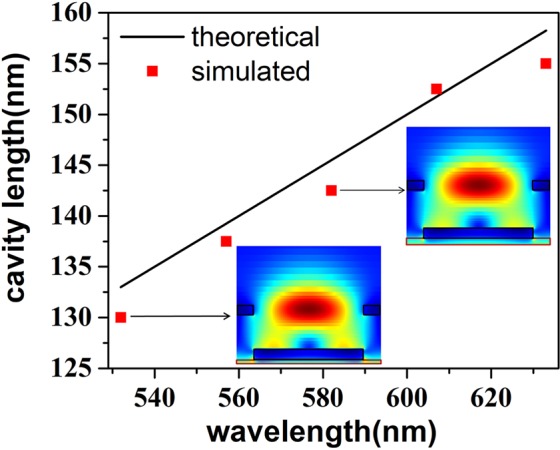
The optimized cavity lengths (with maximum efficiency) in BLM with respect to the working wavelengths. Here, the black line is theoretical prediction by cavity mode and the red square symbols are the simulation results. Two inset figures display the field distributions of optimized cavity at λ = 532 nm and 582 nm.

To further confirm this model, we performed more simulations with respect to different wavelengths searching for the optimized cavity length. The obtained simulation data together with the theoretical prediction are plotted in Fig. 5, which shows quite good agreement with each other. As examples, the field distributions of two optimized cavity modes at *λ* = 532 nm and 582 nm are displayed as inset figures in Fig. 5, which show good consistent with the cavity theory as well as the previous *λ* = 632.8 nm case (see Fig. 4). Thus, we have confidence that the existence of the cavity in the BLM rightly contribute to the improvement of the working efficiencies. It should be mentioned that in a recently published work^33^, a similar type of bilayer plasmonic metasurface was proposed and show a greatly improved efficiency. However, it is recognized from the viewpoint of interference scattering of multipolar meta-atom without any discussion on the cavity effect. We do believe that multipolar scattering would also make contribution in the similar system. However, our work provides a new perspective to understand the enhancement mechanism with a cavity mode, which was well validated by convincing experimental and simulation data.

## Conclusions

In summary, we have demonstrated a bilayer metallic metalens with improved efficiency both in simulation and experiments. The focusing efficiencies can be improved considerably (~2.6 times) compared with conventional single-layer metallic metalens, in which the cavity length can be optimized to reach a maximum improvement in efficiency. Our approach also can simplify the nanofabrication procedure, which is helpful in massive and efficient production. The cavity design enriches the mechanism for designing metasurfaces and is promising for possible dynamic tuning with the external electric field applied on electro-responses inclusions inside the cavity as the double metallic layers can work as the electrodes properly.

## Methods

### Optical measurement setups

In experiments, the focal spots profiles and efficiencies of light focused by metalenses are measured via our home-made optical setup. A He-Ne laser (λ = 632.8 nm) beam is collimated by an objective (10× magnification) with a beam size much larger than the metalens. The collimated beam then passes through a polarizer and a quarter-waveplate to generate circularly polarized light. An objective (50× magnification) is used to image the light focused by metalens for measuring the size of the focal spot and a lens (f = 200 mm) is used to project the image on a CCD camera. Another group of a polarizer and a quarter-waveplate is arranged between the lens and the camera for circular polarization check. In the imaging experiment, the He-Ne laser is replaced by a halogen white-light source and the 1951 United State Air Force (USAF) resolution test chart is used as the imaging target. The beam is focused by the objective onto the target object and the metalens is placed a focal length away from the object and paired with an objective and a lens to form an image on a CCD camera. When measuring the monochromatic image quality, a filter at 630 nm wavelength is inserted before the camera.

### Finite-difference time-domain numerical simulations

Finite-difference time-domain (FDTD) simulations (Lumerical FDTD) are performed to analysis the cavity enhancement mechanism of the BLM. In modeling the unit cells, periodic boundary conditions are defined at the x and y boundaries and perfectly matched layers (PML) at the z boundaries. The refractive indices of Al, SiO_2_ and PMMA are taken from the software and the unit cells are illuminated by a normalized-incident plane wave source with circular polarization in full 3D simulations. The electric-field distributions are obtained via the surface-detectors.
